# Spin‐State Switching of Spin‐Crossover Complexes on Cu(111) Evidenced by Spin‐Flip Spectroscopy

**DOI:** 10.1002/anie.202411865

**Published:** 2024-10-24

**Authors:** Sven Johannsen, Roberto Robles, Alexander Weismann, Karl Ridier, Richard Berndt, Manuel Gruber

**Affiliations:** ^1^ Institut für Experimentelle und Angewandte Physik Christian-Albrechts-Universität zu Kiel 24098 Kiel Germany; ^2^ Centro de Física de Materiales CFM/MPC (CSIC-UPV/EHU) 20018 Donostia-San Sebastián Spain; ^3^ LCC, CNRS and Université de Toulouse, UPS, INP 31077 Toulouse France; ^4^ Faculty of Physics and CENIDE University of Duisburg-Essen 47057 Duisburg Germany

**Keywords:** spin crossover, scanning probe microscopy, single-molecule studies

## Abstract

Spin‐crossover compounds can be switched between two stable states with different magnetic moments, conformations, electronic, and optical properties, which opens appealing perspectives for technological applications including miniaturization down to the scale of single molecules. Although control of the spin states is crucial their direct identification is challenging in single‐molecule experiments. Here we investigate the spin‐crossover complex [Fe(HB(1,2,4‐triazol‐1‐yl)_3_)_2_] on a Cu(111) surface with scanning tunneling microscopy and density functional theory calculations. Spin crossover of single molecules in dense islands is achieved via electron injection. Spin‐flip excitations are resolved in scanning tunneling spectra in a magnetic field enabling the direct identification of the molecular spin state, and revealing the existence of magnetic anisotropy in the HS molecules.

## Graphical abstract

Spin‐state switching of a spin‐crossover complex on Cu(111) induced by injection of tunneling electrons. The spin‐state is readout from the low‐voltage differential‐conductance spectrum: the high‐spin state exhibits magnetic excitation steps evolving with the application of a magnetic field while no spectroscopic feature is observed in the low‐spin state.

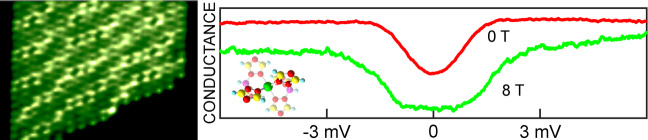

## 1 Introduction

Spin‐crossover (SCO) compounds are transition‐metal complexes that can be switched between a low‐spin (LS) and a high‐spin (HS) state using various external stimuli such as temperature, light, and electrical current.^[1]^ The spin‐state transition is associated with a change of the molecular conformation and of the electronic and optical properties making such systems attractive for applications such as data storage, displays, and sensors,^[2]^ possibly on the scale of single molecules.[^3^, ^4^] Along this line, ultrathin films of molecular SCO compounds have been fabricated by thermal evaporation under vacuum^[5]^ and investigated.[^4^, ^5^, ^6^, ^7^, ^8^, ^9^, ^10^, ^11^, ^12^, ^13^, ^14^, ^15^, ^16^, ^17^, ^18^, ^19^, ^20^, ^21^, ^22^, ^23^] Examples of successful electron and light induced excited spin state trapping (ELIESST, LIESST) have been reported from transport measurements[^24^, ^25^, ^26^, ^27^, ^28^, ^29^, ^30^, ^31^, ^32^, ^33^, ^34^] and scanning tunneling microscopy (STM).[^20^, ^23^, ^35^, ^36^, ^37^, ^38^, ^39^, ^40^, ^41^, ^42^, ^43^, ^44^, ^45^, ^46^, ^47^]

SCO complexes in direct contact with surfaces have attracted much interest but direct evidence of spin‐state switching at the single‐molecule level is difficult to obtain. As the switching of an Fe molecule is not necessarily due to SCO, the absence of switching on analogue Ni or Zn complexes,[^45^, ^46^] where no SCO is expected, provides indirect evidence. Comparisons with the results of density functional theory calculations, which predict different geometries and frontier orbital energies for the high and low spin states have also been used.[^10^, ^45^, ^46^] Finally, the observation of a Kondo resonance, which signals the presence of a localized spin, has been employed.[^23^, ^35^, ^36^] In the case of Fe(II) complexes only the HS state carries a magnetic moment and can lead to this effect. However, the Kondo effect is only observed when the coupling of the molecular spin and the conduction electrons happens to be suitably strong. A spin‐state readout that is directly related to the magnetic moment of the metal‐ion remains desirable.

Here we investigate the SCO complex [Fe(HB(1,2,4‐triazol‐1‐yl)_3_)_2_] on a Cu(111) surface with low‐temperature STM. Individual molecules in large monolayer islands are switched by injecting electrons. Tunneling spectra of the molecules in the HS state exhibit steps that shift in a magnetic field. These inelastic features reflect spin‐flip excitations that are directly linked to the molecular spin states enabling their direct identification.

## 2 Results and Discussion

### 2.1 Self‐Assembly on Cu(111)

**Figure 1:**
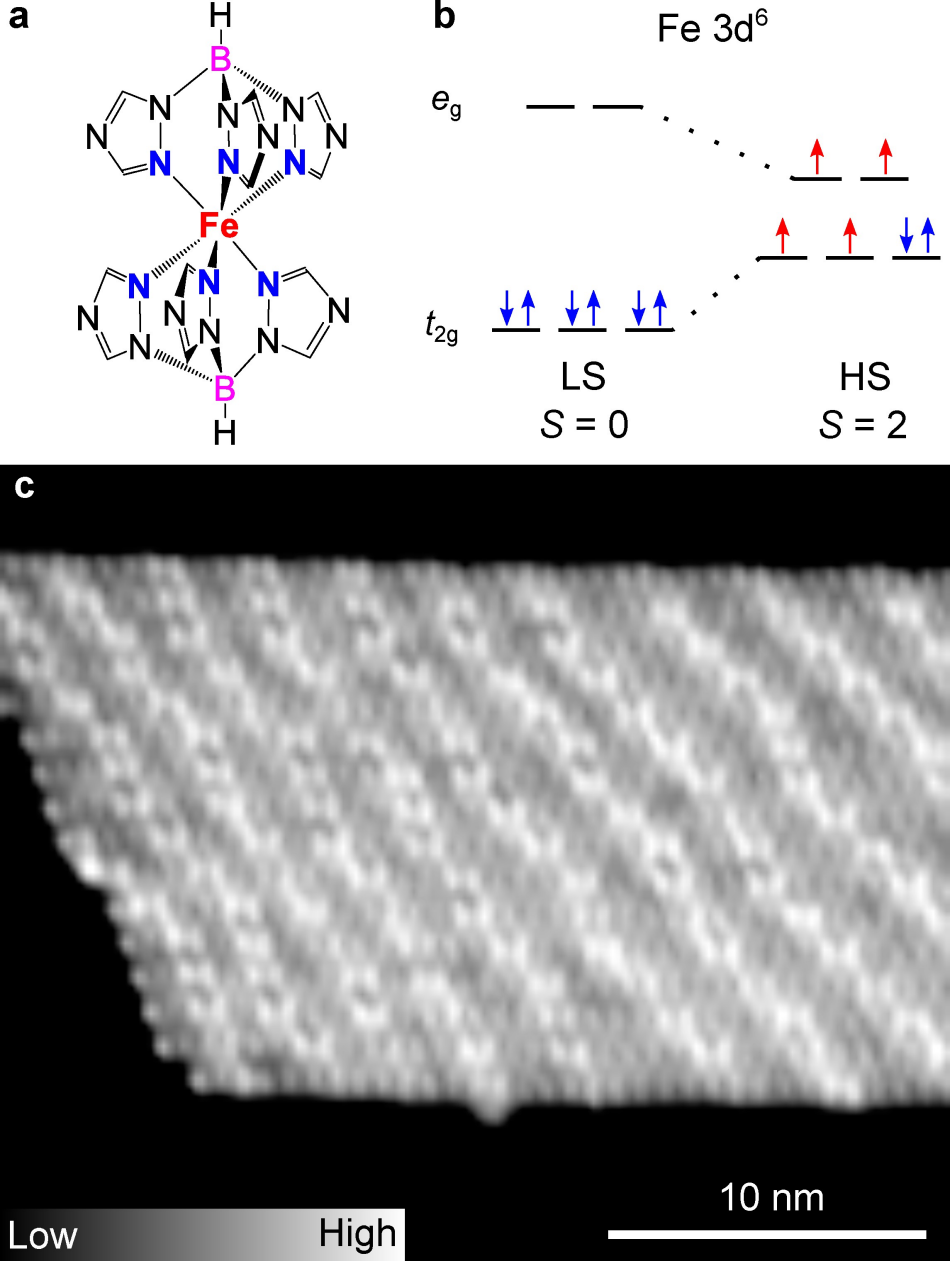
**a**) Molecular structure of the complex [Fe(HB(1,2,4‐triazol‐1‐yl)_3_)_2_]. **b**) Schematic representation of the 3*d* orbitals for an Fe(II) (3*d* ^6^) complex in an octahedral ligand field. Blue (red) arrows indicate the orbital occupations with paired (unpaired) spins for LS and HS states. **c**) Overview STM topograph (0.2 V, 5 pA, 30×25 nm^2^) of a [Fe(HB(1,2,4‐triazol‐1‐yl)_3_)_2_] island on Cu(111). The apparent height of the layer with respect to the Cu substrate is 220 pm. In addition to the molecular contrast, bright stripes with an apparent height of ≈30 pm are observed.

The investigated [Fe(HB(1,2,4‐triazol‐1‐yl)_3_)_2_] complex (Figure 1a) composed of two identical tridentate ligands, exhibits a transition temperature of ≈334 K in the bulk material.^[48]^ As common for Fe(II) complexes in an octahedral ligand‐field geometry, the electronic configuration in the LS and HS state leads to a total spin of *S*=0 and *S*=2, respectively (Figure 1b). Films (20–200 nm) of this compound prepared via sublimation in vacuum are of high quality, crystallize upon exposure to water vapor, and essentially exhibit the same transition temperature as bulk samples.^[49]^ Such (thick) films have been extensively investigated[^50^, ^51^, ^52^] and integrated in electronic^[28]^ and photonic devices.[^53^, ^54^]

Upon sublimation of submonolayer amounts of [Fe(HB(1,2,4‐triazol‐1‐yl)_3_)_2_] on Cu(111), the molecules self‐assemble into large islands, typically ≈20 nm wide and ≈40–80 nm long with an apparent height of ≈220 pm at 0.2 V (Figure 1c). The islands exhibit bright stripes with a periodicity of ≈3 nm. It seems that the molecules have an epitaxial relation with the substrate, as observed for the complex [Fe((3,5‐(CH_3_)_2_Pz)_3_BH)_2_] on Au(111).^[18]^ The stripes are likely due to misfit dislocations to release the strain within the layer caused by lattice mismatch.

**Figure 2:**
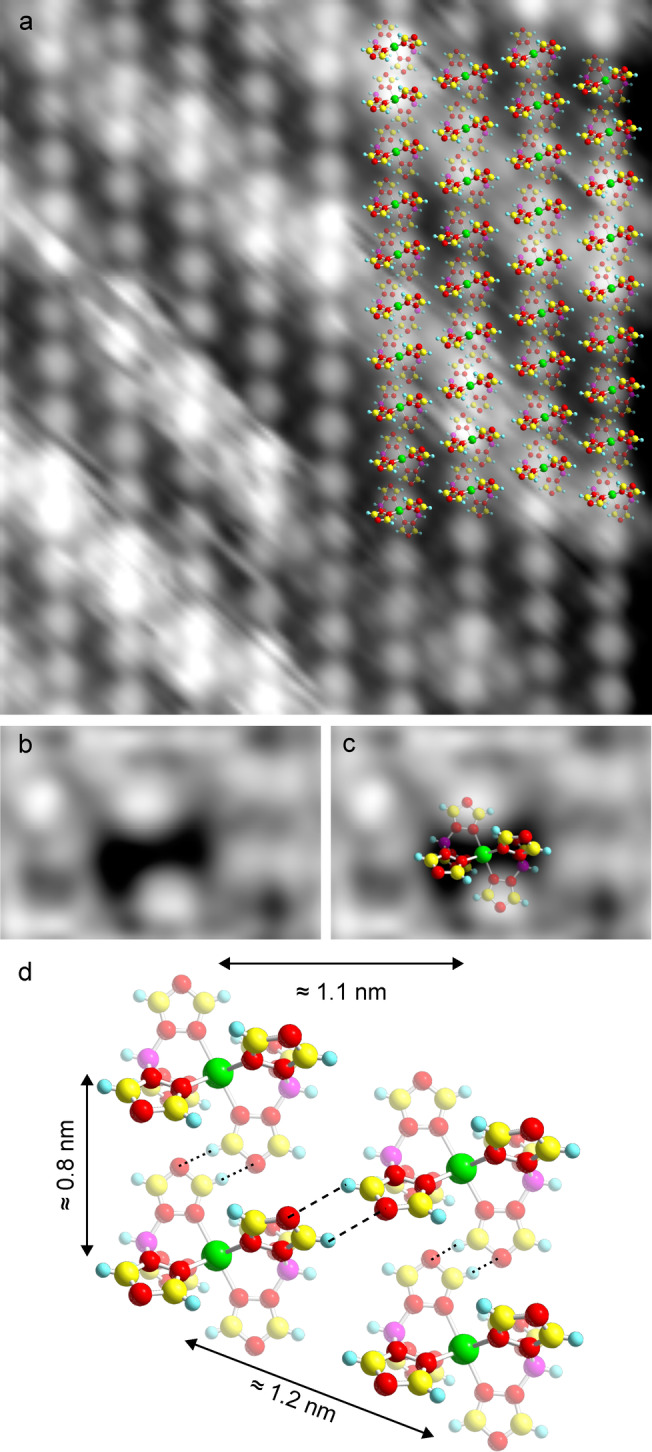
**a**) Topograph of [Fe(HB(1,2,4‐triazol‐1‐yl)_3_)_2_] on Cu(111) with overlaid molecular models (1.6 V, 5 pA, 10×11 nm^2^). **b**, **c**) Zoomed topographs of an area from which a molecule has been picked up (200 mV, 5 pA, 3×2 nm^2^). The remaining depression is horizontally elongated with a lateral extent matching that of the upper two triazole groups of a flat lying molecule. **d**) Proposed arrangement of the molecules on the Cu(111) surface, confirmed by density functional theory calculations. For **a**–**d**, the transparency of the atoms indicates the height relative to the Cu(111) surface with transparent atoms being closer to the substrate. Green, yellow, red, purple, and cyan spheres respectively represent Fe, C, N, B, and H atoms.

Figure 2a shows a smaller area of the molecular layer on Cu(111). A regular arrangement of circular protrusions is observed along with fuzzy areas parallel to the top‐left to bottom‐right diagonal of the image. The protrusions and fuzzy areas correspond to individual molecules and the stripes, respectively (discussed above). To gain more information about the orientation of the molecules, we picked up a molecule from the layer by approaching the tip toward a protrusion and increasing the setpoint current from 5 to 20 pA at *V*=50 mV. After depositing the molecule elsewhere, the remaining hole is observed as an horizontal depression (Figure 2b). Assuming that the image contrast is mainly caused by the upper part of a flat‐lying molecule, we suggest that the orientation of the molecules is as shown in Figure 2c, where the lateral dimensions of the depression match with the upper two triazole groups of a molecule. Picking up further molecules from the same island leads to depressions with the same orientation (SI, Section 5), indicating that all molecules within an island have the same orientation. We therefore propose the molecular structure shown in Figure 2d and in the overlay of Figure 2a.

According to the proposed structure, the molecules presumably form lines along the long direction of the islands with a periodicity of 1.1 nm (Figure 2d). Within each line, two neighboring molecules may be connected via C−H…N bonds between the underlying triazole groups (dotted lines in Figure 2d) leading to a center‐to‐center distance of ≈0.8 nm. Adjacent lines are vertically shifted and most probably stabilized by two C−H…N interactions between the upper triazole groups (dashed lines in Figure 2d). We note that the adsorbed molecule is chiral and the two enantiomers form different stackings leading to two types of islands (SI, Sections 6 and 7). The proposed molecular structure is supported by density functional theory calculations (see below). It should be noted that structures involving both enantiomers, with the experimentally resolved periodicity and orientation of the molecules, lead to spatial overlaps of neighboring molecules and were therefore discarded.

### 2.2 Voltage Dependent Image Contrast

**Figure 3:**
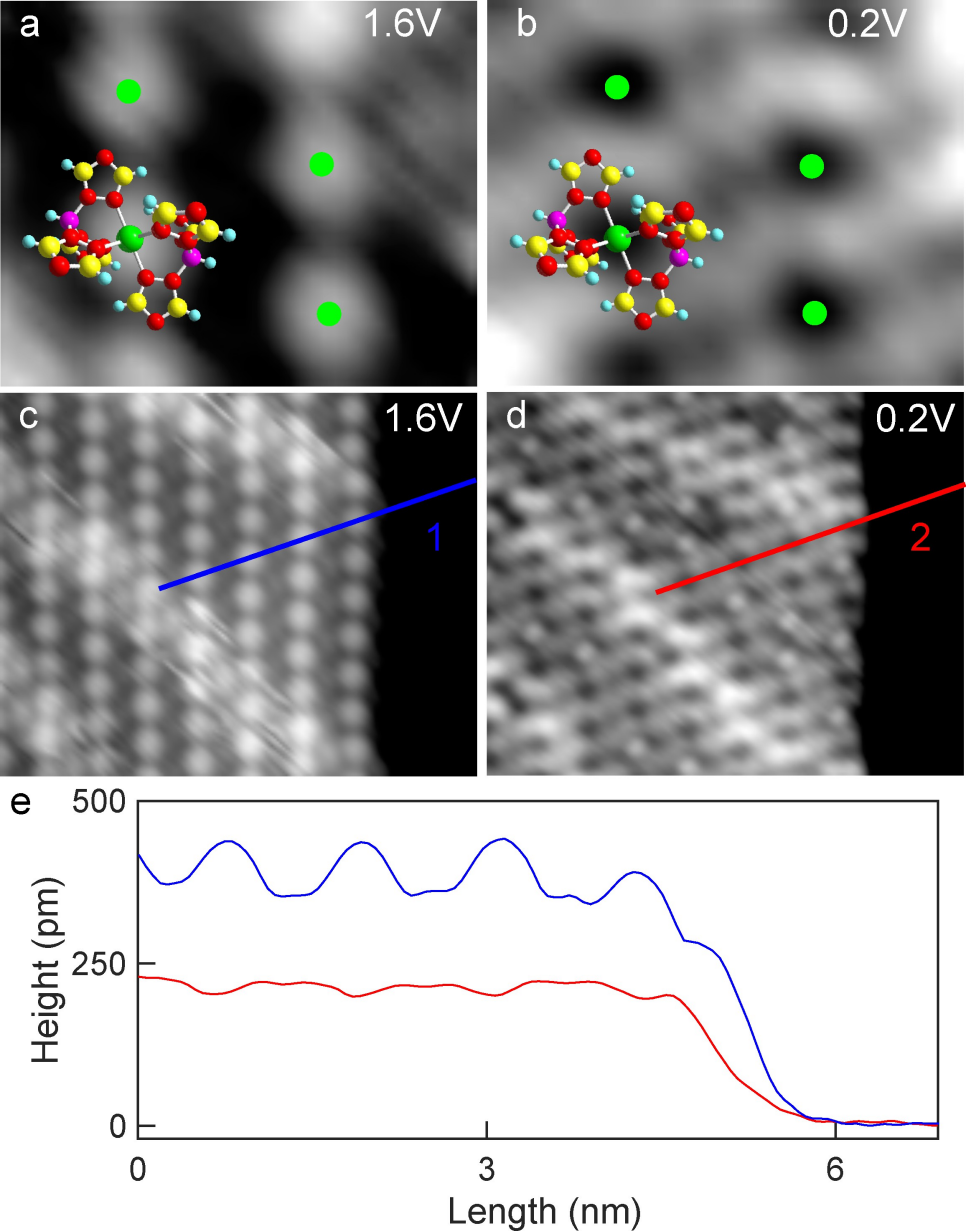
**a**, **b**) Topographs of a 2.6×2.1 nm^2^ area from a molecular island recorded at 1.6 and 0.2 V, respectively with *I*=5 pA. While each [Fe(HB(1,2,4‐triazol‐1‐yl)_3_)_2_] complex is imaged as a single protrusion at 1.6 V, the image contrast is reversed at 0.2 V. The suggested adsorption geometry is indicated by a superposed model and by green dots at the positions of the Fe ion centers. **c**, **d**) Larger topographs (10×8 nm^2^) of an island measured with the parameters used in panels **a** and **b**. At 1.6 V parallel columns of virtually identical protrusions are observed along with diagonal stripes. At 0.2 V the former protrusions, which correspond to single molecules, appear mostly as depressions (or as small protrusions upon switching as detailed below). **e**) Cross‐sectional profiles along the paths indicated by blue and red lines in **c** and **d**. At 1.6 V, the molecules appear approximately twice as high compared to 0.2 V (≈430 vs. ≈220 pm). The plateau at the right side of the height profiles shows the bare Cu(111) substrate.

The appearance of the molecules as circular protrusions at 1.6 V (Figures 3a, c) presented above, drastically changes when the sample voltage is reduced to 0.2 V (Figures 3b, d). At this voltage, the molecules essentially appear as depressions (see Figure 3b, where green dots indicate the positions of the Fe atoms). The change of the sample voltage leads to a reduction of the apparent height by almost a factor 2, from ≈430 pm at 1.6 V to ≈220 pm at 0.2 V (Figure 3e). This drastic modification of the molecular contrast indicates a large non‐geometric contribution to the STM image. The importance of electronic contributions is further evidenced in images of island edges, where the border molecules appear smaller (SI, Section 9), and independently supported by density functional theory calculations (below). One or more unoccupied molecular orbitals are expected to have energies between 0.2 and 1.6 eV. Unfortunately, the acquisition of d*I*/d*V* spectra to confirm the presence of the orbitals failed because of large current fluctuations at voltages exceeding ≈0.5 V, as further discussed below.

### 2.3 Electron‐Induced Switching

**Figure 4:**
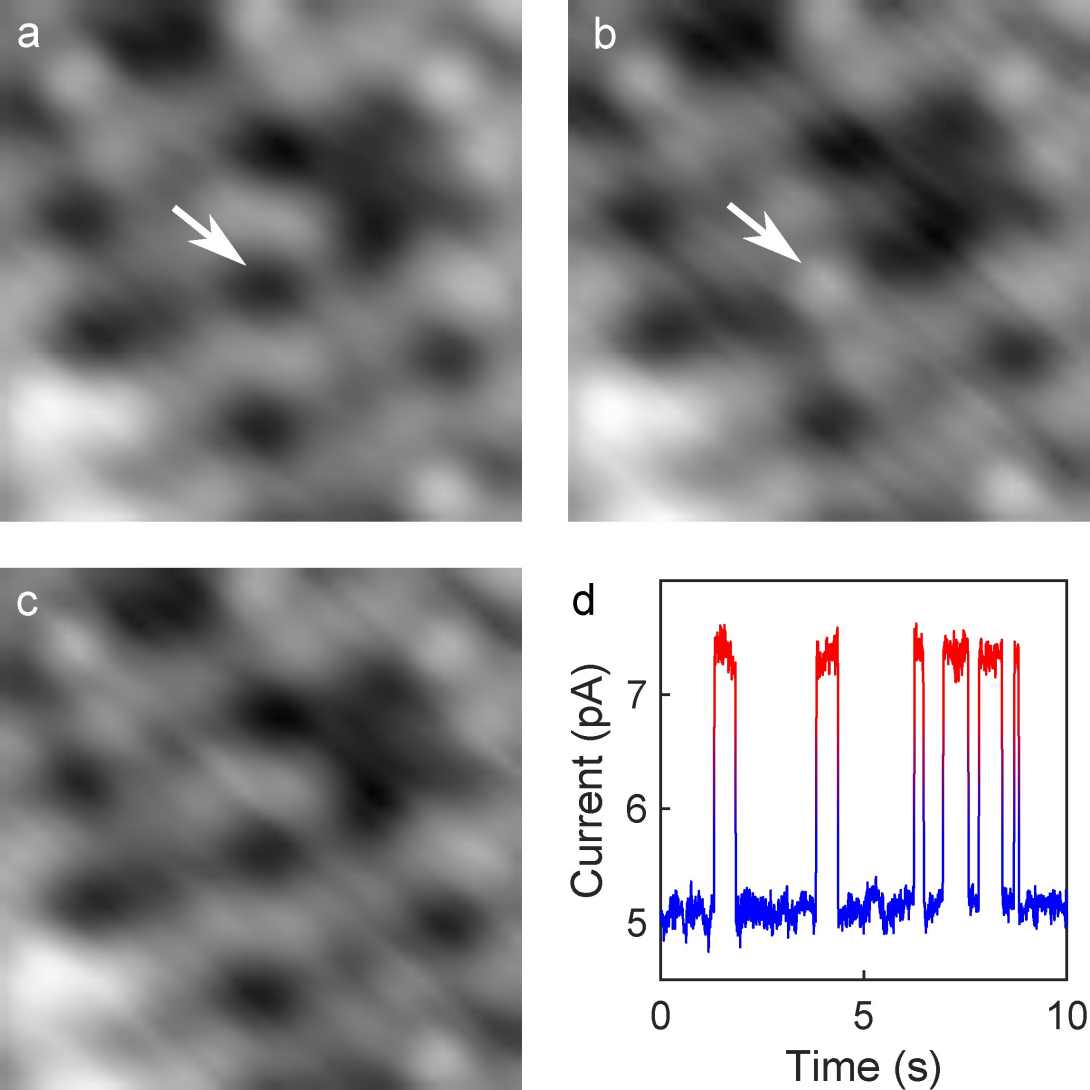
Reversible switching of an individual molecule under the tip. **a**) Topograph (0.2 V, 5 pA, 3×3 nm^2^) of an island with the molecule at the center (white arrow) in its pristine state appearing as a depression. **b**) Same area imaged at 0.2 V after the voltage had been raised to 0.5 V for a few seconds with the tip placed above the central molecule (current feedback enabled). The former depression converted to a small protrusion. **c**) After repeating the manipulation procedure, the center molecule reverted to its original state. **d**) Time series of the tunneling current measured at *V*=0.5 V with feedback disabled. Frequent switching is detected between current levels of ≈5.1 and 7.4 pA. Imaging at low bias revealed that the current levels reflect a depression and a protrusion, respectively.

Switching of the molecules may be induced by injecting electrons at a sample voltage of ≈0.5 V or higher. Figure 4 shows an example. The pristine molecule appears as a depression at 0.2 V as previously described (white arrow in Figure 4a). After having placed the tip above the center of the depression, raising the voltage to 0.5 V for a few seconds, and decreasing it back to 0.2 V, while maintaining the current feedback loop active, the same molecule appears as a small protrusion (Figure 4b). The molecule may then be switched back to its pristine state using the same procedure (Figure 4c). Alternatively, for voltages above 0.5 V, telegraph noise is observed in time series of the tunneling current (feedback loop inactive). The low and high current levels correspond to a molecule appearing as a depression and protrusion, respectively.

**Figure 5:**
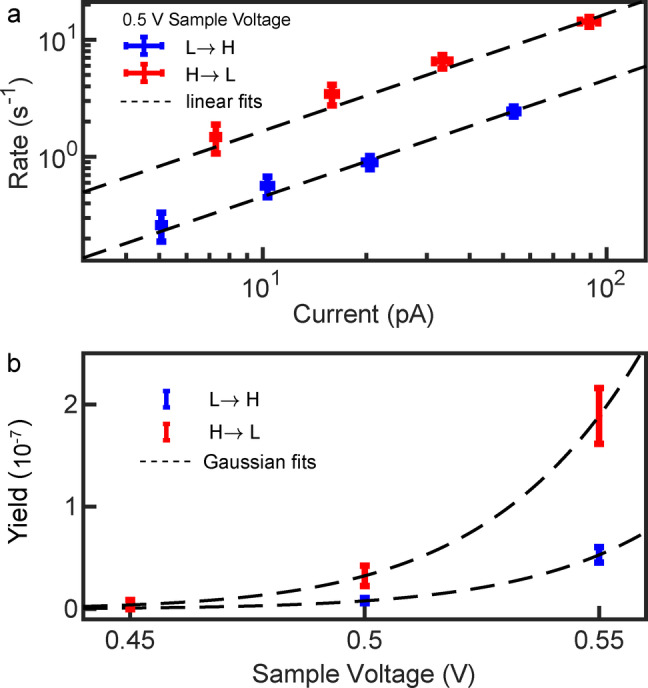
Switching rates and yields. **a**) Rate of switching from the pristine L state to the H state and back as a function of the tunneling current. **b**) Switching yields, defined as the switching rate normalized to the tunneling current, measured at three sample voltages. The uncertainty bars represent one standard deviation.

Telegraph noise of the tunneling current was acquired for different tip‐molecule distances and voltages, from which we can extract the switching rates (see Ref. [46] for details). The switching rates from a low (L) to a high (H) current value and vice versa evolve linearly with the tunneling current (Figure 5a) indicating that each switching event is caused by a single electron (the rate of a multi‐electron process would evolve with *I* ^*N*^ with *N*>1 in contrast to *N*=1 for a single‐electron process). The probability of a tunneling electron to induce the switching is on the order of 10^−7^, comparable to the highest reported yields for SCO molecules on surfaces (up to ≈8×10^−7^ in Ref. [46]). The yield evolves rapidly with the applied voltage (Figure 5b). At 0.45 V, two transitions were observed in a time windows of 50 s (for *I*=5 pA), while at 0.5 V switching is monitored every few seconds for currents as low as 5 pA. This implies that only electrons with an energy ≥0.5 eV can switch the molecules. Interestingly, a similar threshold has been determined for the compound [Fe((3,5‐(CH_3_)_2_Pz)_3_BH)_2_] on Cu(111),^[20]^ suggesting that the two compounds might share a common microscopic switching mechanism.

**Figure 6:**
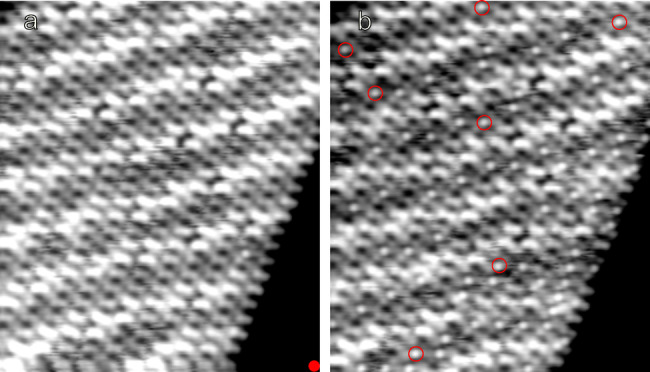
Long‐range switching. **a**) STM image of an island with molecules in the pristine (L) state. 10 pulses of 2 V for 200 ms each (*I* reaching ≈55 pA during the pulses) were applied on the Cu(111) substrate at the position indicated by a red dot. **b**) STM image after the series of pulses. Several molecules switched to the H state, some of which are highlighted with red circles. Switched molecules are observed at distances as far as 21 nm away from the excitation point. This distance represents a lower bound as molecules outside the scanned area may have switched as well. Both images were acquired at 0.2 V, 5 pA (15.6×18.2 nm^2^).

Besides the local, controlled conversion of individual molecules, switching may be remotely triggered at distances up to ≈20 nm (Figure 6). Pulses of 2 V were applied on the Cu(111) surface (red dot in Figure 6a), which lead to the switching of many molecules, some of them encircled in red in Figure 6b. Interestingly, molecules up to ≈20 nm away from the location of the pulses were switched as well. This fairly efficient and long range remote switching is most probably due to hot electrons that propagate along the Cu(111) surface.^[55]^

It should be noted that in islands that have not been exposed to voltages above 0.2 V, the images of all molecules (at 0.2 V, Figure S9) show a depression in their center. This suggests that all molecules are in the same pristine state before manipulation.

### 2.4 Density Functional Theory Calculations

**Figure 7:**
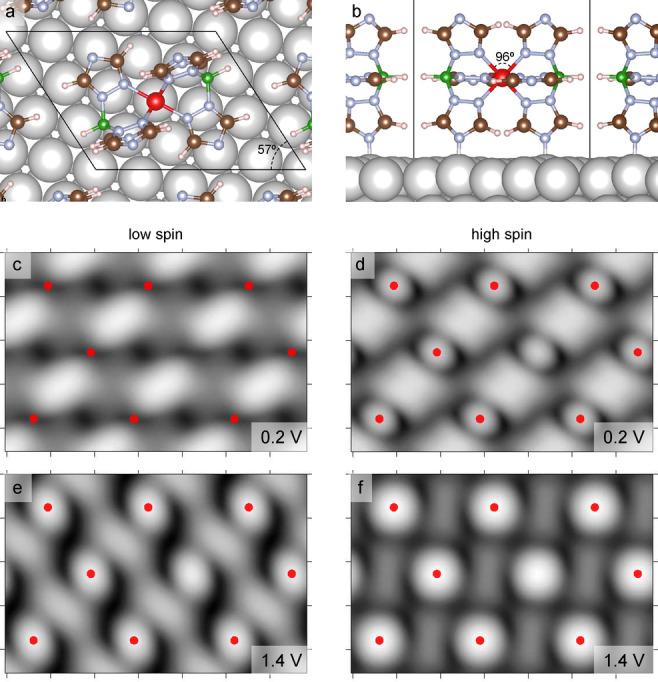
**a**) Top and **b**) side views of the optimized structure of [Fe(HB(1,2,4‐triazol‐1‐yl)_3_)_2_] on a Cu(111) surface. The black parallelogram indicates the unit cell used for the calculation. **c**–**f**) Simulated constant‐current STM images of nine [Fe(HB(1,2,4‐triazol‐1‐yl)_3_)_2_] molecules in the low‐spin and high‐spin states at 0.2 and 1.4 V. Red dots indicate Fe ions. The central molecule is not marked to better visualize the topographic features.

We performed density functional theory (DFT) calculations to better analyze the change of the image contrast with the sample voltage and with the spin state of the molecules. Figure 7a shows the unit cell used for the calculations with dimensions of 1.149×0.904 nm^2^ and an angle of 57° between the lattice vectors, which is close to the experimentally determined unit cell (≈1.2×0.8 nm^2^, 68°). It is worth noting that the DFT calculations show that the adsorption of [Fe(HB(1,2,4‐triazol‐1‐yl)_3_)_2_] on the Cu(111) surface induces a small distortion of the molecule. The Fe−N coordination bonds of the molecule in the gas phase have the same length (1.98 Å for LS and 2.18 Å for HS). Upon adsorption, the molecule is found to be slightly compressed although the Fe−N bonds hardly change (0.01 Å for LS and 0.02 Å for HS). The N−Fe−N angle connecting upper and lower triazole moieties of the HS molecule (Figure 7) reduces from 86° in the gas phase to 83° on the surface, while the N−Fe−N angle linking the two upper triazole groups evolves from 95° to 96° upon adsorption.

The simulated topographs at 0.2 V exhibit a depression at the position of the Fe ion in the LS state (Figure 7c), which turns into a protrusion in the HS state (Figure 7d). This is consistent with our experimental observations in Figure 4. At 1.4 V, molecules in the LS (Figure 7e) and HS (Figure 7f) states appear as large protrusions. At this voltage, the molecules are most probably rapidly switching back‐and‐forth so that the experimental topograph time‐averages the features of both spin states, weighed by the respective residence times.

Further adsorption geometries were considered with different lateral positions of the Fe ion relative to the substrate (top, bridge, hollow sites), and with different azimuthal angles of the molecule relative to the substrate. The different geometries are further discussed in the SI, Section 3, but none of these other geometries satisfactorily reproduces the depression observed in the LS state at 0.2 V.

**Figure 8:**
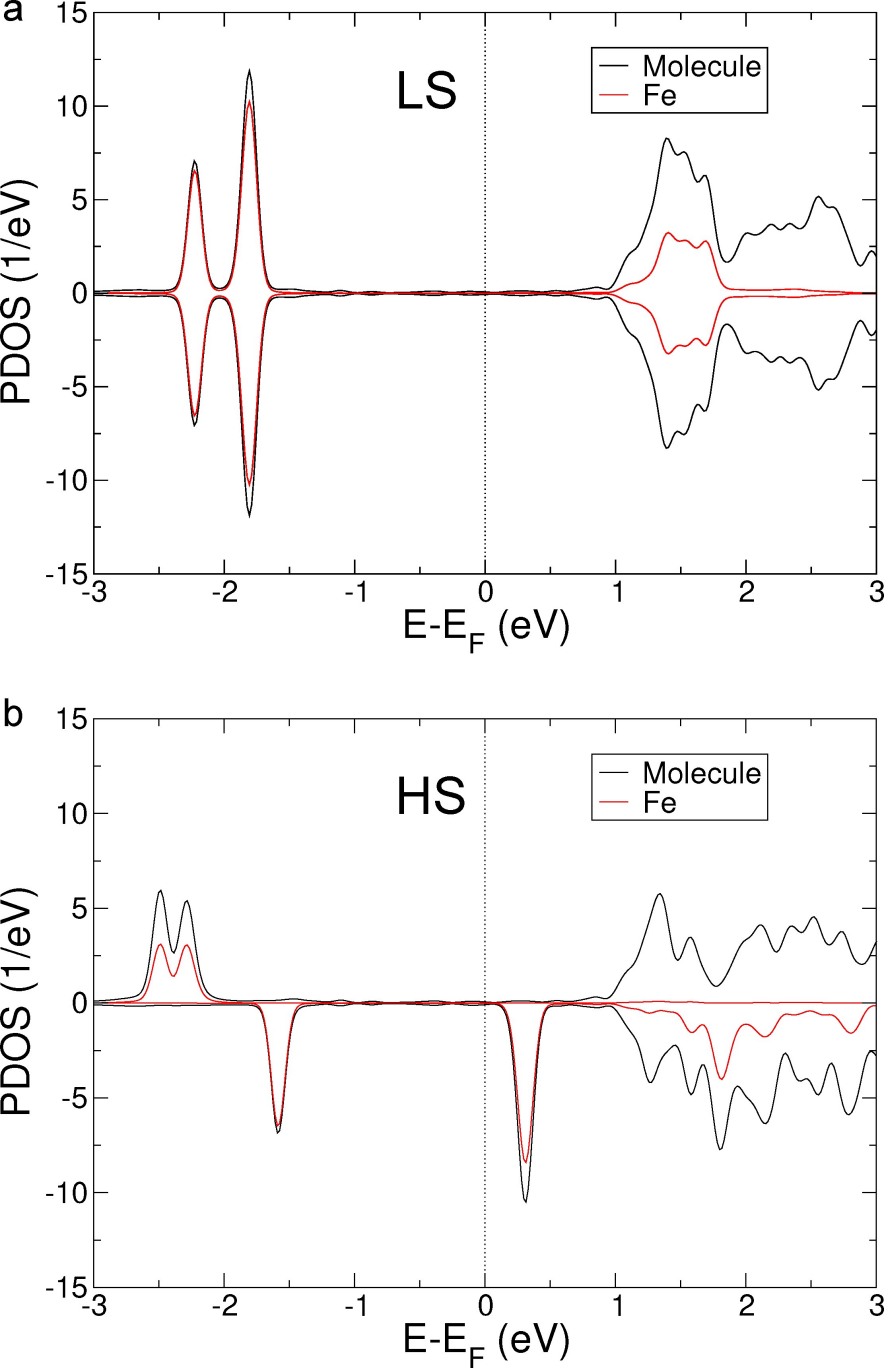
Calculated spin‐resolved projected density of states of [Fe(HB(1,2,4‐triazol‐1‐yl)_3_)_2_] on Cu(111) **a**) in the LS and **b**) in the HS states. The projected densities onto the whole molecule and onto the Fe atom are shown in black and red, respectively. Positive and negative PDOS refer to spin‐up and spin‐down polarizations.

The calculations reveal a LS ground state, which is 176 meV lower in energy than the *S*=2 HS state. Figure 8 shows the projected density of states (PDOS) of the molecule on Cu(111) in the LS and HS states. We observe significant unoccupied PDOS above 1 eV in the LS state, which is consistent with the strong change of apparent height of the molecule upon changing the sample voltage from 0.2 to 1.6 V (Figure 3). In the HS state, the spin up and spin down PDOS are different. The energy gap between the highest occupied and lowest unoccupied molecular orbitals is significantly reduced compared to the LS state. In particular, there is a significant PDOS at low positive voltage with a maximum at ≈0.3 V. This contribution, broad in energy and with a strong localization on the Fe ion, is responsible of the topographic contrast a low voltage: the molecules in the HS state are observed with a protrusion atop the Fe center at low voltages.

### 2.5 Identification of Spin States

**Figure 9:**
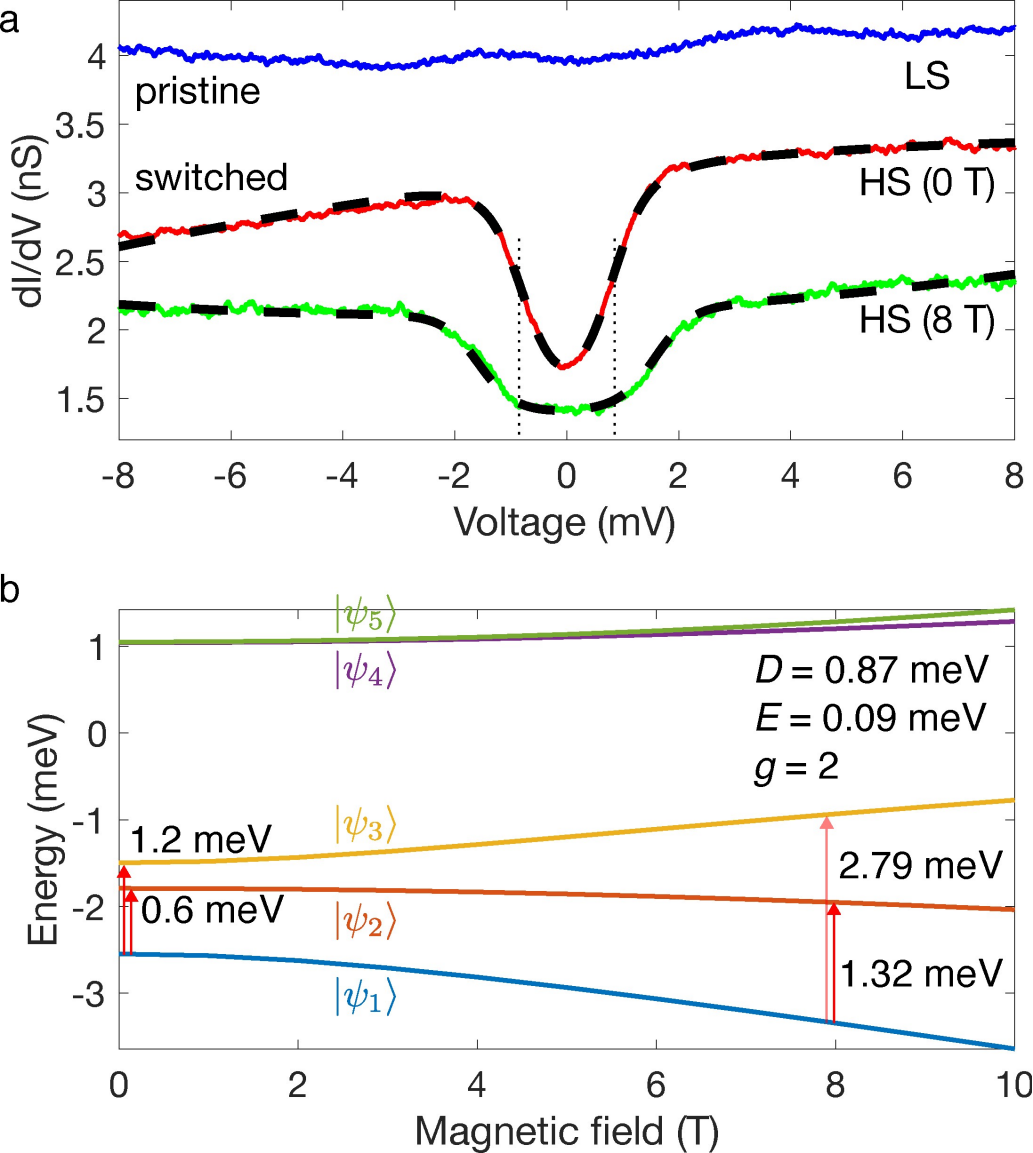
Identification of the spin states via spin‐flip spectroscopy. **a**) d*I*/d*V* spectra acquired atop the center of a molecule in the pristine (blue, LS) and switched (red, HS) states at 1.7 K (without external magnetic field). The spectrum of the switched molecule exhibits a dip consistent with broadened inelastic excitation steps (spin flips). In a magnetic field of 8 T, the steps are shifted to higher energies and have a lower amplitude (green), confirming the magnetic origin of the excitation steps. The spectra of the switched molecule can be fitted together (dashed lines) using the spin Hamiltonian of Eq. (1) with *S*=2, *D*=0.87 meV and *E*=0.09 meV including temperature broadening (*T*=1.8 K) as well as a cubic background. *g* is fixed to 2 to reduce the number of adjustable parameters. Spectra acquired over a pristine molecule under 8 T remain featureless (not shown). The vertical dotted lines highlight the increase of the step energies with magnetic field. The LS and HS (8 T) spectra have been vertically shifted by +1 and −1 nS for clarity. Prior to the voltage sweep, the current feedback was opened at 10 mV and 30 pA. Topographs of the corresponding molecules are shown in the SI, Section 11. **b**) Energies of the eigenstates $\left|\psi_{i}\right\rangle$ as a function of the external magnetic field for *D*=0.87 meV, *E*=0.09 meV, and *g*=2. The vertical red arrows indicate possible transitions at 0 and 8 T via spin‐flip processes with tunnel electrons. The semi‐transparency of the 2.79 meV arrow indicates that the associated change of conductance is small.

High‐resolution d*I*/d*V* spectra of the molecules have been recorded with and without magnetic field. Molecules in the pristine (L) state essentially exhibit no features in the energy range ±8 meV (blue curve in Figure 9a). In contrast, the spectrum of the switched (H) state shows a pronounced dip centered at the Fermi level (red curve in Figure 9a). The dip is consistent with broadened inelastic excitation steps. The energy shift and amplitude decrease of these inelastic steps in a magnetic field of 8 T evidence that these excitations are related to the molecular spin. They are associated to spin flips involving an exchange of angular momentum between the molecule and the tunneling electrons. Based on these observations, and in accordance with the DFT calculations, we ascribe the pristine and switched states to the LS and HS states, respectively.

The presence of magnetic excitation steps at 0 T indicate that the molecular magnetic moment has a preferred orientation in the absence of magnetic field, i.e. magnetic anisotropy is present. We use the following phenomenological spin Hamiltonian to describe the energy of the magnetic states:^[56]^

(1)

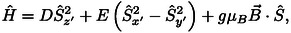




where *D* and *E* are the uniaxial and transverse anisotropy parameters, 
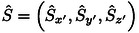
 the spin operator, *g* the Landé *g*‐factor, *μ_B_* the Bohr magneton, and *B* the applied magnetic field perpendicular to the surface. We consider a total spin *S*=2, i.e. the formal spin of the HS state, which leads to an effective magnetic moment of $2\cdot \sqrt{S\left(S+1)\right)}$ ≈4.9 $\mu_{B}$ per molecule comparable with that inferred from magnetometry measurements.^[48]^ The diagonalization of the Hamiltonian of Eq. (1) leads to eigenstates $\left|\psi_{i}\right\rangle$ (i=1...5 for *S*=2) and eigenenergies *E_i_*, from which differential‐conductance spectra are simulated.^[57]^ The parameter set *D*=0.87 meV, *E*=0.09 meV, and *g*=2, with the quantization axis 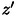 parallel to the surface and perpendicular to $\vec{B}$ , leads to the best agreement with the experimental data (dashed curves in Figure 9). (Fits considering *S*=1 or 3 lead to much poorer agreements.) As discussed in the SI, Section 10, a quantization axis colinear with $\vec{B}$ cannot be excluded from the measurements. The uniaxial anisotropy parameter is very close to *D*=0.9 meV as inferred from X‐ray magnetic circular dichroism measurements of the complex [Fe((3,5‐(CH_3_)_2_Pz)_3_BH)_2_] on Cu(111), which lends further support to the model.

Our DFT calculations with spin‐orbit coupling reveal a magnetic anisotropy of the molecule, on the order of one meV, which is consistent with the experimental results. The calculated anisotropy is found to be sensitive to the environment, and in particular to the interactions with the substrate (SI, Section 4).

The fit of the d*I*/d*V* spectra with the Hamiltonian of Eq. (1) shows the consistency of the data with a molecular spin *S*=2 in the HS state. The exact values of the anisotropy parameters should be confirmed using more detailed measurements in magnetic vector fields or using complementary techniques. With *D*=0.87 meV, *E*=0.09 meV, *g*=2 and in the absence of a magnetic field, the ground state of the system is $\left|\psi_{1}\right\rangle \approx \left|0\right\rangle$ (expressed in the basis 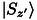 ), *i.e*. with a spin orientation in a plane perpendicular to the surface. The excitation steps then correspond to transitions to $\left|\psi_{2,3}\right\rangle =$ $\left(\left|1\right\rangle \mp \left|-1\right\rangle \right)/\sqrt{2}$ at *E*_2_=0.6 and *E*_3_=1.2 meV, which are experimentally observed as a single broad transition (Figure 9b). Examples of simulated d*I*/d*V* spectra without thermal broadening are shown in the SI, Section 10. The application of a magnetic field perpendicular to the quantization axis leads to a non‐trivial mixing of the states, with for instance, the ground state $\left|\psi_{1}\right\rangle$ having further contributions from the $\left|\pm 1\right\rangle$ states. At 8 T, the largest spin‐flip transition is expected between $\left|\psi_{1}\right\rangle$ and $\left|\psi_{2}\right\rangle$ at 1.32 meV, in addition to a much weaker transition to $\left|\psi_{3}\right\rangle$ at 2.79 meV (Figure 9b). This last transition is not directly resolved but effectively further broadens the spectrum.

It should be emphasized that the identification of the spin states via the observation of magnetic excitations in the d*I*/d*V* spectra is not limited to SCO systems exhibiting magnetic anisotropy. In the case of an isotropic spin system, the Hamiltonian of Eq. (1) would be reduced to the Zeeman term, which splits the 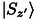 states under a magnetic field. Transitions from $\left|-2\right\rangle$ to $\left|-1\right\rangle$ would then be observed at an energy $g\mu_{B}B$ (≈0.9 meV at 8 T for *g*=2) in d*I*/d*V* spectra.

## 3 Conclusion

We investigated the molecular SCO complex [Fe(HB(1,2,4‐triazol‐1‐yl)_3_)_2_] adsorbed on a Cu(111) surface with low‐temperature scanning tunneling microscopy. The complexes self‐assemble into an ordered structure forming large islands. The molecules are efficiently switched between the LS and HS states by injecting electrons. This may be realized locally by placing the tip above a targeted molecule with a mild voltage (≥0.5 V) or by injecting more energetic electrons (*V*≈2 V) into the substrate. The latter procedure allows the remote switching of molecules in a radius of at least 20 nm from the excitation point. Spectra acquired atop the center of HS molecules reveal excitation steps that change in an applied magnetic field, while no features are found for LS molecules. These observations, supported by DFT calculations, enable an unambiguous spin‐state identification of the adsorbed molecules. The inelastic steps, which correspond to magnetic excitations of the HS molecule, also show that the magnetic moment of the molecule has a preferred orientation. Besides enabling a clear identification of the spin states, these results suggest that manipulation of the quantum spins of the SCO complexes may be feasible.

## Conflict of Interests

The authors declare no conflict of interest.

4

## Supporting information

As a service to our authors and readers, this journal provides supporting information supplied by the authors. Such materials are peer reviewed and may be re‐organized for online delivery, but are not copy‐edited or typeset. Technical support issues arising from supporting information (other than missing files) should be addressed to the authors.

Supporting Information

## Data Availability

The data that support the findings of this study are available from the corresponding author upon reasonable request.
