# Supplementary material for: Spin‐State Switching of Spin‐Crossover Complexes on Cu(111) Evidenced by Spin‐Flip Spectroscopy
Source: Angew Chem Int Ed Engl. 2024 Oct 24;63(51):e202411865. doi: 10.1002/anie.202411865 (PMC11627136; doi:10.1002/anie.202411865)
Supplement: Supplementary file 1 — Supporting Information [file ANIE-63-e202411865-s001.pdf]

Angewandte Chemie
Eine Zeitschrift der Gesellschaft Deutscher Chemiker

Supporting Information

Spin-State Switching of Spin-Crossover Complexes on Cu(111) Evidenced by Spin-Flip Spectroscopy

*S. Johannsen, R. Robles, A. Weismann, K. Ridier, R. Berndt, M. Gruber**

Supporting Information: Spin-State Switching of Spin-Crossover Complexes on Cu(111) Evidenced by Spin-Flip Spectroscopy

Sven Johannsen,† Roberto Robles,‡ Alexander Weismann,† Karl Ridier,¶ Richard Berndt,† and Manuel Gruber*,§

†*Institut für Experimentelle und Angewandte Physik, Christian-Albrechts-Universität zu Kiel, 24098 Kiel, Germany*

‡*Centro de Física de Materiales CFM/MPC (CSIC-UPV/EHU), 20018 Donostia-San Sebastián, Spain*

¶*LCC, CNRS and Université de Toulouse, UPS, INP, 31077 Toulouse, France*

§*Faculty of Physics and CENIDE, University of Duisburg-Essen, 47057 Duisburg, Germany*

E-mail: manuel.gruber@uni-due.de

1 Experimental details

Synthesis The [Fe(HB(1,2,4-triazol-1-yl)$_3$)$_2$] powder was synthesized following the description of Ref. S1. From a CHN elemental analysis of the powder, we find 29.53 % , 2.69 %, and 51.21 % of respectively C, H, and N, which is very close to the expected values (29.55 %, 2.89 %, and 51.68 %).

STM The Cu(111) surface was prepared by cycles of Ar ion bombardment (1.5 keV) and annealing to 500 °C. [Fe(HB(1,2,4-triazol-1-yl)$_3$)$_2$] was sublimated from a heated crucible ($\approx$ 210 °C). STM tips were electrochemically etched from W wire and annealed *in vacuo*. Experiments were carried out in ultrahigh vacuum mostly with a STM operated at $\approx$ 4.6 K (Createc). dI/dV spectra were acquired at 1.7 K with a Unisoku USM1300 STM. The shown dI/dV spectra are obtained from numerical derivation of the current, followed by a convolution with a Gaussian for low-pass filtering.

2 Computational method

Density functional theory calculations were performed using the VASP code.[S2] Core electrons were treated using the projector augmented-wave method.[S3,S4] A plane wave basis set was used with an energy cutoff of 500 eV. The generalized gradient approximation in the PBE form[S5] was used as exchange and correlation functional. Van der Waals interactions were treated with the Tkatchenko-Scheffler method.[S6] The description of 3d Fe electrons was improved by using the GGA+U method[S7] with $U_{\text{eff}} = U - J = 2$ eV. The Brillouin zone was sampled using a $(3 \times 3 \times 1)$ k-point mesh. In order to have a reasonable computational time the experimental unit cell was approximated by relatively small unit cells containing just one molecule. Two different unit cells were considered. Both have a dimension of 1.149 $\times$ 0.904 nm^2. The first option (first two cases in Figure S2) has an angle of 123º, while the second option (last three cases in Figure S2) has an angle of 117º. The Cu(111) surface was simulated using a 5-layer slab. The positions of the molecules and of the first three Cu layers were optimized until forces were below 0.01 eV/Å. Magnetic anisotropy energies were calculated by total energy differences of self-consistent calculations after including spin-orbit coupling.[S8] For the simulation of STM images we used the STMpw code,[S9] which implements the Tersoff and Hamann theory[S10] following the method by Bocquet *et al.*[S11] Charge transfers and magnetic moments were determined by performing Bader analyses.[S12] Images of the structures were generated using the VESTA program.[S13]

To check our method we performed calculations of the [Fe(HB(1,2,4-triazol-1-yl)$_3$)$_2$] complex in the gas phase. In the calculation, a LS ($S = 0$) and two HS solutions can be obtained, one with $S = 1$ and the second with $S = 2$. For Fe(II) in an octahedral environment, the LS configuration is expected to be the ground state, while the HS($S = 2$) configuration is expected to be the next in energy. We have calculated the three configurations as a function of the U_{eff} parameter (Figure S1). As expected, the magnetic configurations get stabilized as we increase U_{eff}. For $U_{\text{eff}} = 0$ eV, the HS ($S = 1$) configuration is more stable than the HS ($S = 2$), contrary to expectations. At the other end, for $U_{\text{eff}} = 3$ eV, HS ($S = 2$) is more stable than the LS configuration,

Table S1: Distances in Å between the central Fe atom and the N atoms, involved in the coordination bonds, of one of the tridentate ligands. The experimental values are taken from S. Rat *et al.*[S1] For the calculated values (gas phase), a $U_{\text{eff}} = 2\,\text{eV}$ was used.

(Å)	LS (exp)	HS (exp)	LS	HS ($S = 1$)	HS ($S = 2$)
Fe-N_1	1.9822(11)	2.137(4)	1.977	2.143	2.181
Fe-N_2	1.9754(11)	2.143(4)	1.977	1.963	2.181
Fe-N_3	1.9758(11)	2.149(4)	1.977	2.143	2.181

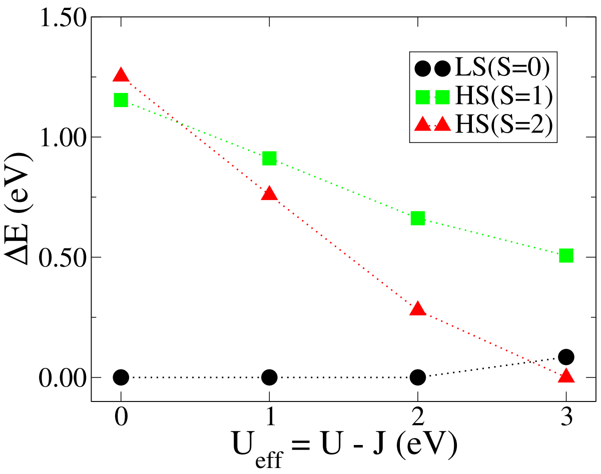

Figure S1: Energy differences of the magnetic configurations of the complex in the gas phase as a function of the U_{eff} parameter. For each value of U_{eff}, the energies are referred to the most stable configuration.

also at odds with the expected behavior. Between these two limits, we selected $U_{\text{eff}} = 2\,\text{eV}$, for which the ordering of the solutions is as expected and the energy difference between LS and HS ($S = 2$) is 0.28 eV. This energy difference ΔE is compatible with experimental results. The transition temperature of the complex is $T_{1/2} \approx 338\,\text{K}$ (Ref. S14) and the change of entropy ΔS is typically between 0.4 and 0.8 meV K^{-1} for Fe(II) SCO complexes.[S15] We therefore expect an energy difference $\Delta E \approx \Delta H = T_{1/2}\Delta S$ ranging between 0.14 and 0.28 eV.

In Table S1, we show relevant calculated distances for $U_{\text{eff}} = 2\,\text{eV}$ and we compare them with experimental results.[S1] We observe that the distances are larger for the HS case than for the LS case, in agreement with the experimental results. The deviations in calculated bond lengths with respect to the experiment are below 0.3 % in the LS state and below 2.1 % in the HS state. Finally, the experimentally determined Fe environment is relatively isotropic for both the LS and HS states. In the calculation the correct isotropic environment is observed for LS and for HS with a $S = 2$ spin state, but not for the $S = 1$ spin state, which further discards this latter spin state as the observed one.

3 Simulated STM images

We considered different adsorption configurations for the molecular layer. Some of the calculated geometries and simulated constant-current STM topographs are shown in Figure S2.

To characterize the adsorption site of the molecule we use the position of the Fe atom over the surface (top, bridge or hollow) and the azimuthal angle. This angle is defined as the angle between the long axis of the molecule (which goes through the Fe atom and the B atom at the right of the image) with respect to the $[1\bar{1}0]$ direction of the Cu(111) surface. For the LS state the topographic images at low (0.2 V) and high (1.4 V) voltages are quite different. The reason can be traced back to the electronic states, which can be seen in the PDOS shown in Figure S3. At 0.2 V the LS configuration shows no states, while at higher voltages (≈1.4 V) there are molecular states. Therefore the image of the molecule at low voltages is due to the tunneling through the tails of the states at higher energies, while at high voltages the image is due directly to the tunneling through the molecular states. However, since the Fe states are typically due to the more localized d states, they have little contribution at 0.2 V and the Fe atom is usually darker at low voltages than at high voltages. In contrast, for the HS state there is a prominent Fe peak at low voltages and the Fe position is always bright, both at low and at high voltages. This behavior is also seen in the experiment and is key for the easy detection of the switching from HS to LS. It is worth noting that the PDOS is quite similar for all the considered cases.

The adsorption energies of the calculated geometries were calculated as $E_{\text{ad}} = E[mol] + E[Cu(111)] - E[mol@Cu(111)]$, where $E[mol]$ is the total energy of the molecule, $E[Cu(111)]$ is the total energy of the surface, and $E[mol@Cu(111)]$ is the total energy of the whole system. The adsorption energies are 2.34, 2.23, 2.50, 2.50 and 2.63 eV for $\Phi = 34°, 36°, 11°, 8°$, and $-35°$, respectively. Since an approximate unit cell was used for the calculations, the intermolecular interactions are not perfectly described, which affects the total energy. In turn, the adsorption energy in this case is not suitable for selecting the best geometry. Instead, we used the agreement between the measured and calculated STM topographs as a criterion. The geometry $\Phi = 34°$ (top) is the only one that leads to topographs at 0.2 V with

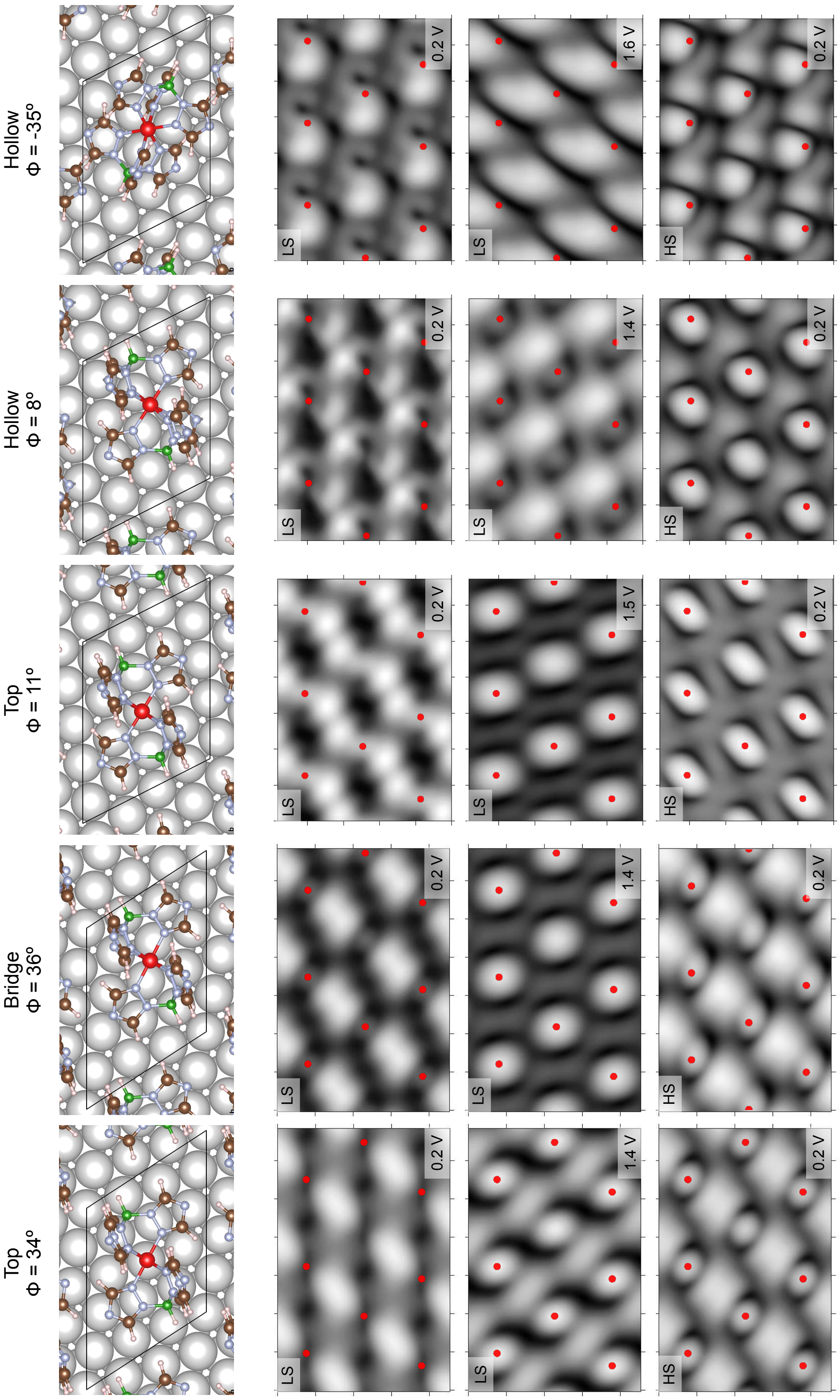

Figure S2: Structure and simulated constant current STM topographies for different geometries considered in the calculations. The geometries are characterized by the position of the Fe atom (top, bridge or hollow) and the azimuthal angle. The first row shows the geometry; the second (third) row shows the simulated STM images for the LS state at 0.2 V ($\approx$ 1.4 V); and the fourth row shows the simulated STM images for the HS state at 0.2 V. The red disks indicate the position of the Fe atoms. Note that the central molecule is not marked to better visualize the topographic features.

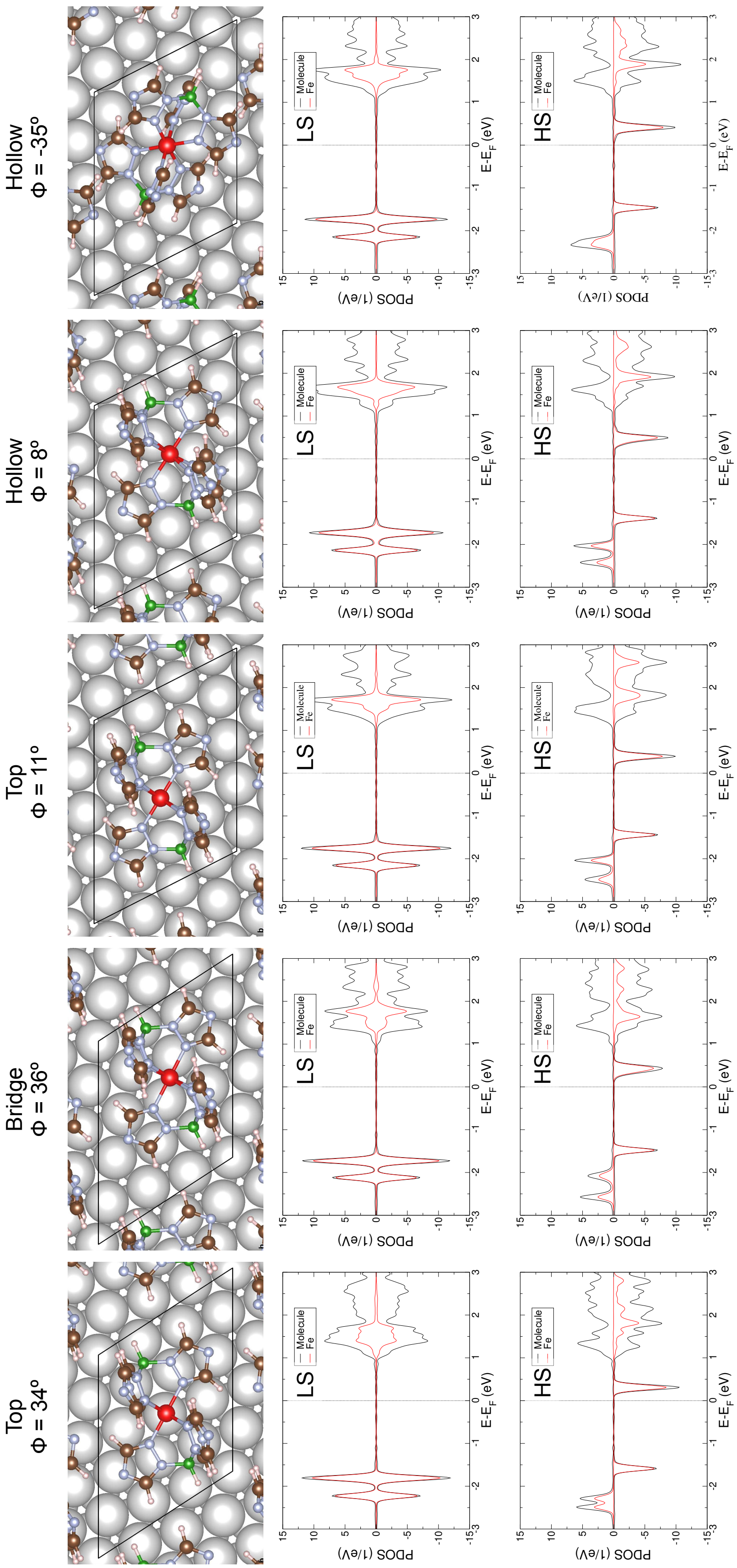

Figure S3: (top) Calculated geometries of [Fe(HB(1,2,4-triazol-1-yl)$_3$)$_2$] on Cu(111) and corresponding projected density of states in the (middle) low-spin and (bottom) high-spin states for various orientations and adsorption sites of the molecule relative to the Cu(111) substrate. The red and black curves correspond to the densities of states projected on the Fe and the molecule, respectively.

depressions at the position of the Fe ions, as observed in the experiments. It is therefore considered further in the main manuscript.

4 Magnetic anisotropy energies

We performed calculations including spin-orbit coupling imposing the spin to be aligned along different directions, thereby obtaining magnetic anisotropy energies. We then computed D and E parameters as[S16]

$$D = \frac{2H_{z'} - (H_{x'} + H_{y'})}{S(2S-1)} \quad \text{(S1)}$$

and

$$E = \frac{H_{x'} - H_{y'}}{S(2S-1)}, \quad \text{(S2)}$$

where z' is along an easy or hard axis of the molecule, H_i is the extra energy cost to align the spin along the axis i, and S is the total spin ($S = 2$).

Table S2: Calculated magnetic anisotropies of different adsorption geometries of [Fe(HB(1,2,4-triazol-1-yl)$_3$)$_2$] on Cu(111). It should be noted that negative D value indicates an easy axis, while a positive value corresponds to an hard axis anisotropy. The values for the molecule in the gas phase are given for comparison. The values of D and E are given in meV.

Φ	ads. site	easy/hard axis	D	E
34	top	in plane	−0.46	0.08
11	top	out of plane	−0.53	0.03
-35	hollow	in plane	+0.37	0.03
8	bridge	in plane	−0.43	0.09
8	hollow	in plane	−0.51	0.08
-13	bridge	in plane	−0.50	0.06
36	bridge	in plane	−0.51	0.03
gas phase			0.70	0.02

For [Fe(HB(1,2,4-triazol-1-yl)$_3$)$_2$] relaxed in the gas-phase, the calculations reveal an easy magnetization plane with the z' hard axis along the long axis of the molecule, passing through the B atoms. The corresponding D and E values are 0.70 and 0.02 meV, respectively. Upon adsorption forming a layer on Cu(111) the magnetic anisotropy changes depending on the adsorption details, as can be seen in Table S2). In most configurations we get an easy axis. For the case of the adsorption at the top site with an azimuthal angle of 34º, the easy axis is parallel to the surface and perpendicular to the long axis of the molecule.

A unit cell with one molecule was used to significantly reduce the computational cost. In turn, we expect deviations of the calculated intermolecular interactions compared to the experimental case. Nevertheless, the calculated anisotropy and the corresponding uniaxial anisotropy constant D are on the order of a meV, which is consistent with the experimental results.

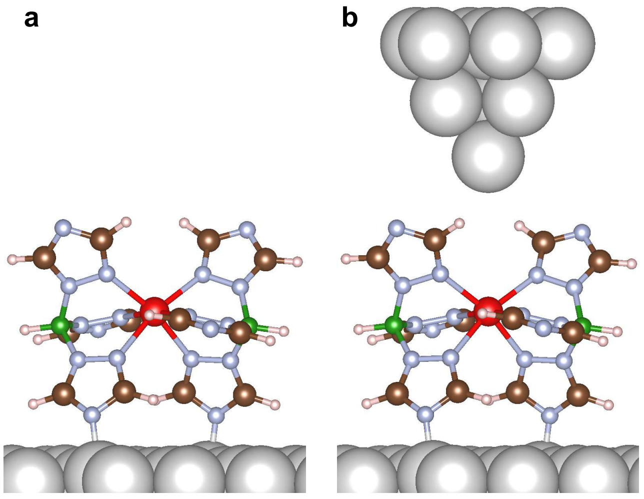

Figure S4: Side view of the relaxed [Fe(HB(1,2,4-triazol-1-yl)$_3$)$_2$] molecule forming a layer on Cu(111) (Φ = 11º) **a** without and **b** with a tip composed of 11 Cu atoms. The center of the lowest atom of the tip is 11 Å away from last atom-layer plane of the substrate.

The spectroscopy measurements were made very close to the molecule, with an estimated tip-substrate distance of ≈ 10 Å (distance between the center of the lowest tip atom and the surface plane intersecting the centers of the uppermost atoms). We therefore performed calculations including a STM tip as shown in Figure S4b. The presence of the tip in the vicinity of the molecule is found to marginally affect the structure of the molecule and the magnetic anisotropy is essentially unchanged. The D and E values evolve from −0.53 and 0.03 meV (out-of-plane easy axis) without the tip to −0.67 and 0.07 meV with the 11 Cu-atom tip.

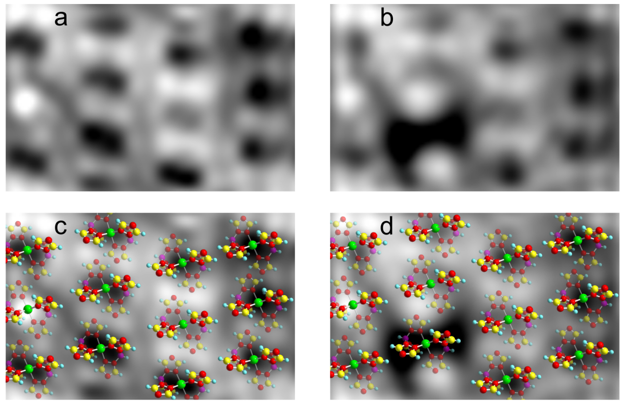

Figure S5: STM images **a** before and **b** after removal of a molecule (200 mV, 5 pA, 2.5 nm high). Topographs **c** and **d** are the same as respectively **a** and **b**, with overlaid molecular models.

5 Molecule removal from an island

Figure S5 shows a series of images illustrating the removal of single molecules from an island. For picking up a molecule, the tip is positioned above the targeted molecule and the tunneling parameters are adjusted to 50 mV and 5 pA. The current feedback remained active during the entire procedure. The tip is then approached toward the molecule by increasing the current to 20 pA. After typically 50 ms, the current increased to ≈ 65 pA and the feedback responded by retracting the tip by ≈ 180 pm. The tip was then indented in the Cu(111) substrate to remove the attached molecule from the tip. As discussed in the main text, the orientation of the depressions left behind indicates the orientation of the molecules within the layers.

We removed up to 6 molecules from a single island as illustrated in Figure S6 for four molecules. All resulting depressions exhibit the same orientation. In particular, we verified that molecules belonging to adjacent rows and columns have the same orientation.

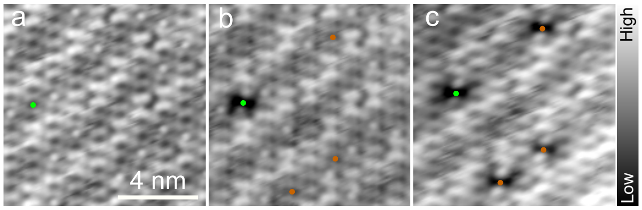

Figure S6: **a**–**c** Series of constant-current topographs of [Fe(HB(1,2,4-triazol-1-yl)$_3$)$_2$] on Cu(111) (200 mV, 5 pA, 10×10 nm^2) illustrating the evolution of a layer as single molecules are removed. The procedure described in the text was applied to the molecule marked with a green dot in **a**, which leads to a depression in the molecular layer after the manipulation (shown in **b**). The molecule removal procedure was then repeated on the molecules marked with an orange dot, leading to the image shown in **c**. The color scales cover **a** 105 pm and **b**–**c** 100 pm.

6 Adsorption-induced chirality

We assume that the molecules lie flat on the surface with the B-Fe-B axis parallel to the surface. A triazole group from each of the two scorpionate ligands makes the contact to the surface (see for instance Figure S7a). Viewed along the B-Fe-B axis, the angle between the triazole groups in contact with the substrate is approximately 60°. The configuration shown in Figure S7b is obtained by rotating the molecule by −60° around the B-Fe-B axis. An azimuthal rotation of the molecule displayed in Figure S7a around the axis perpendicular to the surface cannot lead to that in Figure S7b (see also top views in Figures S7c–d). In other words, the adsorption of the molecule on the surface induces a chirality.

The depression left behind upon the removal of a molecule provides information on the orientation of the molecule defined by the upper triazole groups (opaque atoms in Figures S7c–d), which is the same for all molecules within an island. The periodicity of the molecular structure is directly inferred from the STM images. Attempts to reproduce the regular structure observed in Figure 2 of the manuscript by using the enantiomer shown in Figure S7b, and with a combination of both enantiomers failed due to unphysical partial overlap of the molecules. Only the structure composed of solely the enantiomer shown in Figures S7a,c led to a reasonable result, the one displayed in Figure 2 of the manuscript.

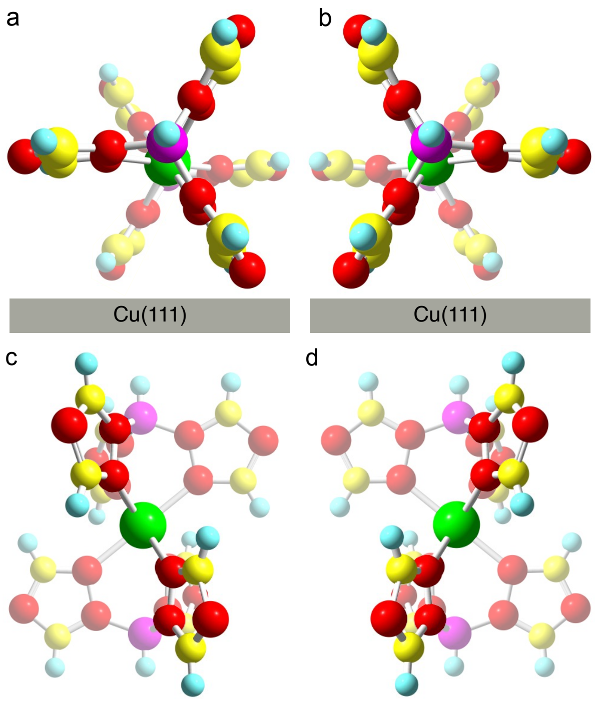

Figure S7: **a**–**b** View of the [Fe(HB(1,2,4-triazol-1-yl)$_3$)$_2$] molecule along the B-Fe-B axis adsorbed on a Cu(111) surface. From **a** to **b** the molecule is effectively rotated by −60° around the B-Fe-B axis. The two configurations correspond to two adsorption-induced enantiomers. **c**–**d** Corresponding top views of the configurations. The transparency of the atoms indicates the distance from the observer. Opaque atoms are closer than semi-transparent atoms.

7 Chirality of islands

As the chirality is induced by adsorption, both enantiomers are expected to be present on the surface. If an island is indeed composed of a single enantiomer, different islands will be present on the surface. This is indeed the case. As detailed in the main text, the islands exhibit stripes, which make them chiral. For instance, the island of Figure S8a has stripes going from the top left to the bottom right. Azimuthal rotation of that island cannot reproduce the island shown in Figure S8b. The removal of molecules from these islands reveals dif-

ferent orientations of the depressions (Figures S8c–d), and hence different orientations of the molecules.

The island shown in Figure S8a is equivalent to that shown in Figure 2 of the manuscript, with the molecular structure shown in Figure S8g. Attempts to reproduce the island shown in Figure S8b using the same enantiomer failed due to unphysical overlaps. Instead, the structure shown in Figure S7h made with the other enantiomer leads to a good match. The two structures are equivalent in terms of the molecule-molecule interactions but are chiral.

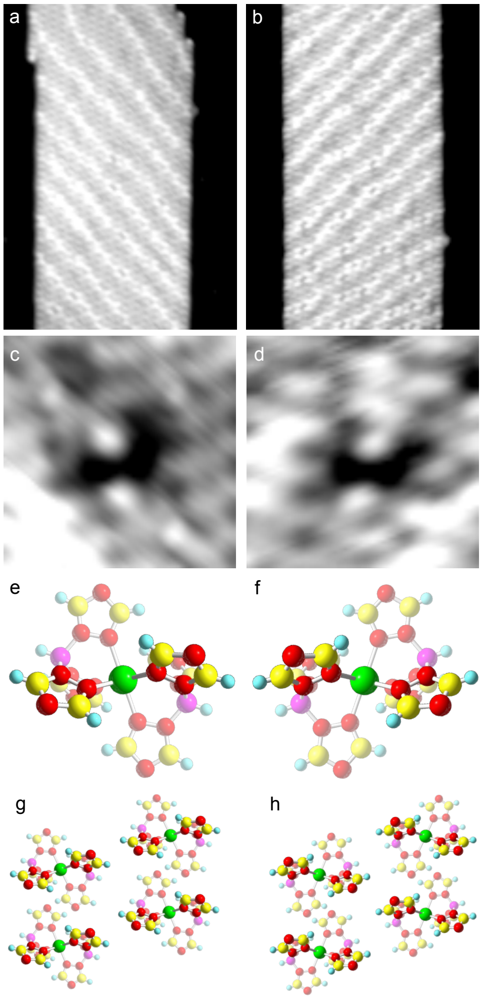

Figure S8: **a**–**b** STM images displaying the chirality of the islands. The island in **a** exhibits stripes from the top left to the bottom right. An azimuthal rotation of **a** cannot reproduce the pattern in **b**, which was observed on the same sample. **c** and **d** Depressions after removal of a molecule from the islands in **a** and **b**, respectively. The molecular structures of island **a** and **b** may be reproduced by using the enantiomers **e** and **f**, respectively. The corresponding molecular structures are shown in **g** and **h**.

8 Island prior to manipulation

The surface of a fresh sample was usually imaged at ≈ 1 V to locate regions of interest with molecular islands. In addition, we shaped the tip (*e. g.* via voltage pulses) on the Cu(111) relatively close to islands. Both of these operations can lead to the uncontrolled switching of molecules. Figure S9 shows an area of the sample, which has been probed by voltages only up to 0.2 V. On such images, we do not observe molecules appearing with a protrusion in the center.

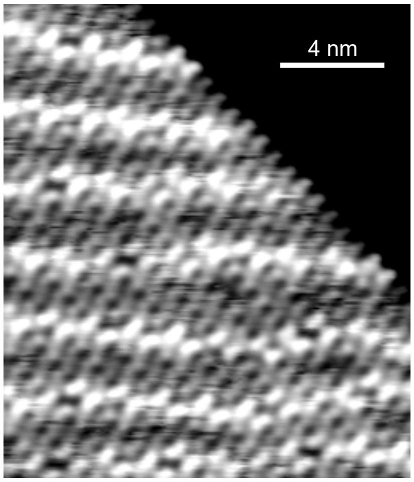

Figure S9: Topograph of a molecular island acquired at 0.2 V. Care was taken to not expose the area to large voltages prior to imaging.

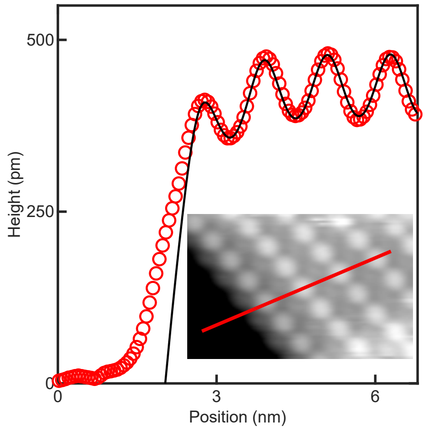

Figure S10: Cross-sectional profile of a [Fe(HB(1,2,4-triazol-1-yl)$_3$)$_2$] island edges along the line shown in the topograph in the inset ($V = 1.6$ V, $I = 5$ pA). Red circles show experimental data. The black line results from a simple model of a tip with limited lateral resolution. No attempt was made to fit the data at low apparent heights because the large vertical excursion of the tip of the STM implies that the unknown three-dimensional tip structure must be taken into account.

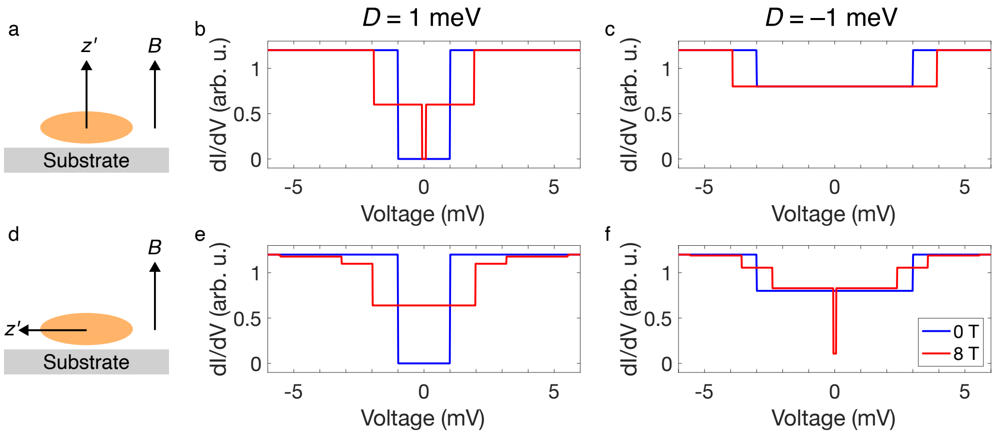

Figure S11: **a** Illustration of a molecule (orange) adsorbed on a substrate (gray). The quantization axis z' of the molecule is perpendicular to the surface and collinear with the external magnetic field B. Simulated dI/dV spectra with **b** $D = +1$ meV and **c** $D = -1$ meV with an effective temperature of 0 K using Equation 1 of the main text. Similar simulations for **e** $D = +1$ meV and **f** $D = -1$ meV when the quantization axis z' is in the plane, perpendicular to B, as shown in **d**. For each set of parameters, a spectrum for $B = 0$ T (blue) and $B = 8$ T (red) are shown. $E = 0$ and $g = 2$ for all calculated spectra.

9 Reduced height of edge molecules

Molecules at the edge of an island appear ≈ 55 pm lower than molecules within islands at 1.6 V (see for instance the left column in the inset of Figure S10). Red circles in Figure S10 shows a cross-sectional profile of an island edge. The height difference may be understood from a simple model that takes into account the lateral extension of the tip. The limited lateral resolution implies that the current above the center of a molecule is increased by the contributions of the neighbors, which are fewer for a molecule at an edge. Adding these current contributions with exponentially decaying weight the experimental data may be qualitatively reproduced (black curve in Figure S10).

10 Simulated dI/dV spectra

The experimental spin-flip spectra on a HS molecule suggest a single excitation step (symmetric about the Fermi level) under a magnetic field of 0 T. The excitation step(s) under 8 T is at higher energy and the amplitude of the excitation is reduced. Figure S11 shows simulated spectra for various configurations of the magnetic anisotropy. The spectrum shown in Figure S11e has the best agreement with the experimental observations, suggesting that the molecule exhibit an in-plane magnetic anisotropy, perpendicular to the substrate (the easy anisotropy plane is perpendicular to z' in Figure S11d).

The spectrum shown in Figure S11b is apparently not reproducing the experimental results. Indeed, the 8 T spectrum exhibit a further low-energy excitation at $\approx 70\,\mu$eV. However, at the temperature of the measurements of 1.7 K, those features would be smeared out due to the associated decreased energy resolution. Therefore, an easy anisotropy plane parallel to the surface cannot be discarded. Future measurements with a vector magnet field, or with other spectroscopic methods, are required to unambigously determine the anisotropy geometry of the adsorbed [Fe(HB(1,2,4-triazol-1-yl)$_3$)$_2$] complex on Cu(111).

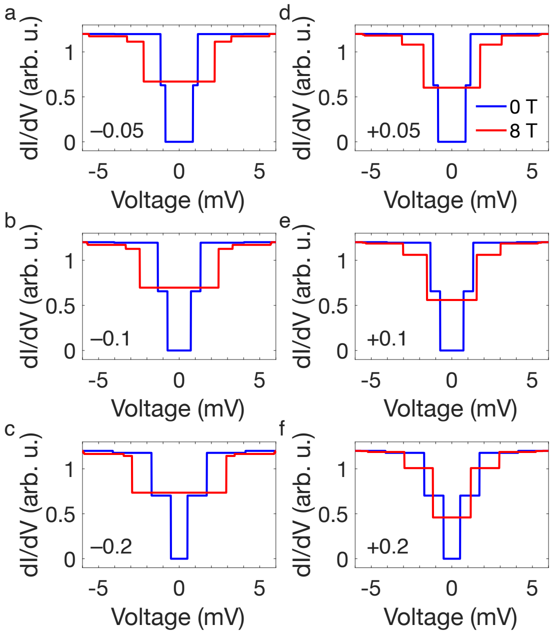

Figure S12: Simulated dI/dV spectra for $D = +1$ meV, 0 K, the quantization axis z' parallel to the surface (see Figure S11d) and for different values of E indicated in each panel in meV.

Figure S12 shows calculated spectra for a configuration as illustrated in Figure S11d, for $D = +1$ meV and various values of the parameters E. $E \neq 0$ essentially leads to more transitions in the spectra, calculated for a temperature of zero Kelvin.

11 Additional information to dI/dV spectra

Figure S13 shows STM topographs along with the tip positions for the acquisition of the differential-conductance spectra shown in Figure 9 of the main text. It should be noted that the tunneling conditions for acquiring such spectra, *i. e.* 10 mV and 30 pA, are optimal for the removal of the molecule as discussed in Section 5, which then required tip shaping to continue the experiments. As such, the success rate of low-voltage spectroscopy measurements is on the order of 1 % and effectively prevented us to acquire all the spectra on the same molecule.

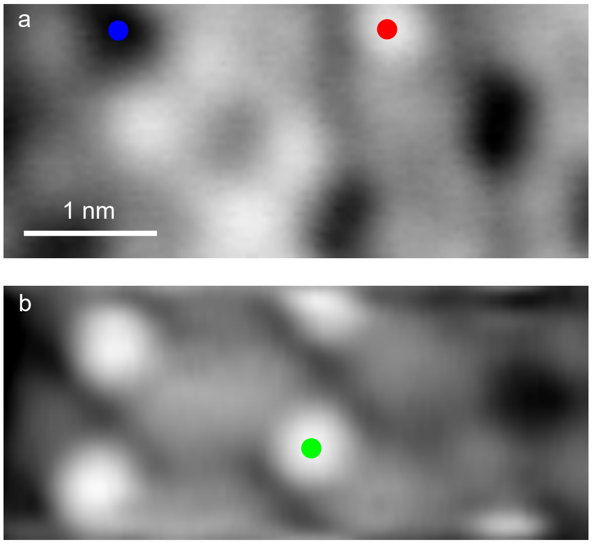

Figure S13: Topographs measured at 0.2 V under magnetic fields of **a** 0 T and **b** 8 T. The blue (pristine, 0 T), red (switched, 0 T), and green (switched, 8 T) dots indicate the tip positions for the spectra shown in Figure 9. The size of the images is $3.5 \times 1.5\,\mathrm{nm}^2$.

12 Comparison of the samples used with the two different instruments

The spectroscopic measurements were acquired on a different instrument and at a different temperature than the rest of the data. Figure S14 compares the images of molecular islands acquired with the two instruments. The different samples had comparable coverages leading to comparable sizes of islands.

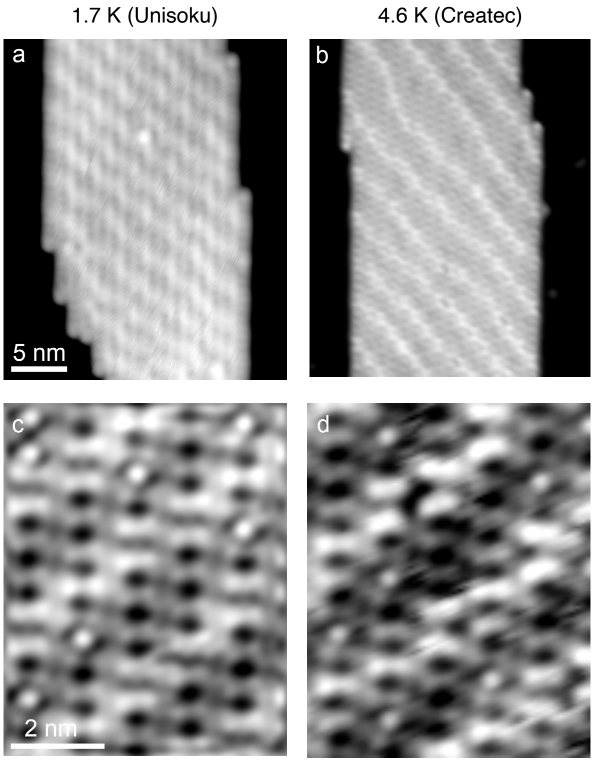

Figure S14: STM images acquired with **a**, **c** with the Unisoku instrument at 1.7 K and with **b**, **d**, the Createc instrument at 4.6 K. The sizes of the images are **a**, **c** $25 \times 30\,\mathrm{nm}^2$ and **b**, **d** $6 \times 7.5\,\mathrm{nm}^2$.

References

(S1) Rat, S.; Ridier, K.; Vendier, L.; Molnár, G.; Salmon, L.; Bousseksou, A. Solvatomorphism and structural-spin crossover property relationship in bis[hydrotris(1,2,4-triazol-1-yl)borate]iron(II). *CrystEngComm* **2017**, *19*, 3271–3280.

(S2) Kresse, G.; Furthmüller, J. Efficient iterative schemes for ab initio total-energy calculations using a plane-wave basis set. *Phys. Rev. B* **1996**, *54*, 11169–11186.

(S3) Blöchl, P. E. Projector augmented-wave method. *Phys. Rev. B* **1994**, *50*, 17953.

(S4) Kresse, G.; Joubert, D. From ultrasoft pseudopotentials to the projector augmented-wave method. *Phys. Rev. B* **1999**, *59*, 1758.

(S5) Perdew, J. P.; Burke, K.; Ernzerhof, M. Generalized gradient approximation made simple. *Phys. Rev. Lett.* **1996**, *77*, 3865.

(S6) Tkatchenko, A.; Scheffler, M. Accurate molecular van der Waals interactions from ground-state electron density and free-atom reference data. *Phys. Rev. Lett.* **2009**, *102*, 073005.

(S7) Dudarev, S. L.; Botton, G. A.; Savrasov, S. Y.; Humphreys, C. J.; Sutton, A. P. Electron-energy-loss spectra and the structural stability of nickel oxide: An LSDA+U study. *Phys. Rev. B* **1998**, *57*, 1505.

(S8) Steiner, S.; Khmelevskyi, S.; Marsmann, M.; Kresse, G. Calculation of the magnetic anisotropy with projected-augmented-wave methodology and the case study of disordered $Fe_{1-x}Co_x$ alloys. *Phys. Rev. B* **2016**, *93*, 224425.

(S9) Lorente, N.; Robles, R. STMpw v1.0b2 (Zenodo), can be found under https://doi.org/10.5281/zenodo.3581159. 2019.

(S10) Tersoff, J.; Hamann, D. R. Theory of the scanning tunneling microscope. *Phys. Rev. B* **1985**, *31*, 805–813.

(S11) Bocquet, M.-L.; Lesnard, H.; Monturet, S.; Lorente, N. In *Computational Methods in Catalysis and Materials Science*; Santen, R. A. v., Sautet, P., Eds.; Wiley-VCH Verlag GmbH & Co. KGaA, 2009; pp 199–219.

(S12) Tang, W.; Sanville, E.; Henkelman, G. A grid-based Bader analysis algorithm without lattice bias. *J. Phys. Condens. Matter* **2009**, *21*, 084204.

(S13) Momma, K.; Izumi, F. VESTA 3 for three-dimensional visualization of crystal, volumetric and morphology data. *J. Appl. Crystallogr.* **2011**, *44*, 1272–1276.

(S14) Ridier, K.; Bas, A.-C.; Zhang, Y.; Routaboul, L.; Salmon, L.; Molnár, G.; Bergaud, C.; Bousseksou, A. Unprecedented switching endurance affords for high-resolution surface temperature mapping using a spin-crossover film. *Nat. Commun.* **2020**, *11*, 3611.

(S15) Nicolazzi, W.; Bousseksou, A. Thermodynamical aspects of the spin crossover phenomenon. *C. R. Chim.* **2018**, *21*, 1060–1074.

(S16) Choi, D.-J.; Robles, R.; Gauyacq, J.-P.; Ternes, M.; Loth, S.; Lorente, N. Structural and magnetic properties of $FeMn_x$ chains ($x = 1 - 6$) supported on Cu_2N/Cu(100). *Phys. Rev. B* **2016**, *94*, 085406.
